# TB programme outcomes in South Fly District, Papua New Guinea, were maintained through COVID-19

**DOI:** 10.5588/pha.24.0020

**Published:** 2024-12-01

**Authors:** M. Bauri, S. Vaccher, T. Marukutira, K.L. Huang, A. Murray, G. Chan, L. Morris, M. Boga, S.M. Graham, N. Wuatai, S.S. Majumdar

**Affiliations:** ^1^ Western Provincial Health Authority, Daru, Papua New Guinea;; ^2^ Burnet Institute, Melbourne, VIC, Australia;; ^3^ University of Melbourne, Department of Paediatrics, Royal Children’s Hospital, Melbourne, VIC, Australia.

**Keywords:** COVID-19, tuberculosis, Papua New Guinea

## Abstract

**SETTING:**

An established response to an outbreak of drug-resistant TB (DR-TB) on Daru Island, South Fly District (SFD), Western Province, Papua New Guinea (PNG).

**OBJECTIVE:**

To describe and evaluate the trends in TB case notification disaggregated by demographic and clinical characteristics, programmatic interventions for TB and COVID-19 and treatment outcomes in 2017–2022.

**DESIGN:**

A cohort study of routinely collected programmatic data of all patients registered for TB treatment in SFD comparing pre-COVID (2017–2019) to COVID (2020–2022) periods.

**RESULTS:**

Of the 3,751 TB cases registered, 19.6% had DR-TB, and the case notification rate was 1,792/100,000 for Daru and 623/100,000 for SFD. There was a 29.2% reduction in case notifications from 2019 to 2021, with recovery in 2022. During COVID, the healthcare workforce was adversely impacted, and active TB case-finding was stopped. During COVID, compared to pre-COVID, bacteriological confirmation increased (62.3% to 71.9%), whereas rates of child TB notifications (11.6% to 9.1%), pulmonary TB (60.8% to 57.4%) and DR-TB (20.7% to 18.6%) decreased. High rates of treatment success were maintained for both drug-susceptible (86.5%) and DR-TB (83.6%).

**CONCLUSION:**

Health systems strengthening and community engagement before COVID likely contributed to resilience and mitigated potential impacts on TB in this remote and resource-limited setting. Case notifications remain very high, and additional interventions are needed to interrupt transmission.

## Abstract (fr)

**CONTEXTE:**

Une réponse établie face à une épidémie de TB résistante aux médicaments (DR-TB, pour l’anglais « *drug-resistant TB* ») sur l'île de Daru, dans le district de South Fly (SFD), province occidentale, Papouasie Nouvelle Guinée (PNG).

**OBJECTIF:**

Présenter et évaluer les évolutions concernant la notification des cas de TB, en tenant compte des caractéristiques démographiques et cliniques, ainsi que des interventions programmatiques liées à la TB et au COVID-19, et des résultats thérapeutiques entre 2017 et 2022.

**MÉTHODES:**

Une analyse de cohorte des données programmatiques recueillies systématiquement auprès de tous les patients inscrits pour le traitement de la TB dans le SFD, en comparant les périodes précédant la COVID (2017–2019) à celles durant la COVID (2020–2022).

**RÉSULTATS:**

Parmi les 3 751 cas de TB recensés, 19,6% étaient atteints de DR-TB. Le taux de notification des cas s'élevait à 1 792 pour 100 000 habitants à Daru et à 623 pour 100 000 habitants pour SFD. Entre 2019 et 2021, le nombre de notifications a chuté de 29,2%, avant de connaître une reprise en 2022. Pendant la pandémie de COVID-19, le personnel de santé a été affecté négativement, entraînant l'arrêt de la recherche active des cas de TB. Durant cette période, la confirmation bactériologique a augmenté, passant de 62,3% à 71,9%, tandis que les taux de notification de la TB infantile ont diminué (de 11,6% à 9,1%), tout comme ceux de la TB pulmonaire (de 60,8% à 57,4%) et de la DR-TB (de 20,7% à 18,6%). Les taux de réussite du traitement sont restés élevés pour la TB sensible aux médicaments (86,5%) et pour la DR-TB (83,6%).

**CONCLUSION:**

Le renforcement des systèmes de santé ainsi que l'implication des communautés avant la pandémie de COVID-19 ont sans doute joué un rôle crucial dans la résilience et la réduction des effets potentiels sur la TB dans cette région isolée et à ressources limitées. Les notifications de cas demeurent très élevées, et des mesures supplémentaires sont indispensables pour freiner la transmission.

TB is a leading cause of morbidity and mortality globally, with an estimated 4,000 deaths each day.^1^ Papua New Guinea (PNG) is classified as a high-burden country for both drug-susceptible (DS) and multidrug/rifampicin-resistant TB (MDR/RR-TB) by the WHO. The estimated incidence of TB in PNG in 2022 was 432/100,000 people, and TB mortality in people without HIV was 44/100,000 people.^1^ The emergence and transmission of drug-resistant TB (DR-TB) in PNG is a major challenge.^2,3^ DR-TB is more complex to diagnose and treat than DS-TB, requiring additional resources, which places stretched health systems under further strain.^4,5^

In the South Fly District (SFD) of Western Province, a significant outbreak of MDR/RR-TB on Daru Island has been the focus of an emergency response since 2014.^4,6,7^ We have previously reported on enhanced case detection, improved treatment outcomes, stabilisation of transmission from 2014 to 2017 and initiation of community-based contact investigation and management.^6,8,9^ The case notification rate was 736/100,000 in SFD in 2017 and much higher in Daru. Drivers of TB transmission include environmental and socioeconomic factors such as overcrowded settlements, food insecurity and household poverty.^10,11^

The COVID-19 pandemic has caused disruptions to healthcare service delivery, particularly in low and middle-income countries with less resilient health systems.^12,13^ The impact on TB services includes reduced case detection and worse treatment outcomes, resulting in unprecedented setbacks to global TB control and almost half a million excess deaths from TB from 2020 to 2022.^1,14^ PNG suffered significant disruption to health care and services through workforce impacts, diversion of resources and disrupted systems such as diagnostics and supplies.^12,13^ We describe the changes to the TB response before and after the onset of the COVID-19 pandemic and evaluate TB case detection and treatment outcomes in SFD, PNG.

## METHODS

### Study design and population

A cohort study of routinely collected programmatic data of all people registered with DS and DR-TB in South Fly District, PNG, was conducted from 1 January 2017 to 31 December 2022.

### Study setting

Western Province had a population of approximately 315,273 in 2021^15^ and is divided into three administrative districts: North Fly District, Middle Fly District and SFD (population: 84,435). The provincial capital is Daru, in SFD, a small and densely populated island with an estimated population of 19,397 in 2021 living predominantly in overcrowded settlements.^15,16^ The population fluctuates throughout the year.^11^ The remoteness and limited road access make accessing and delivering health and essential services extremely challenging. Daru TB basic management unit (BMU) diagnoses and treats DS and DR-TB for all SFD and parts of Middle Fly District if services are unavailable. It contains the Daru Provincial Hospital (DPH), the provincial referral hospital.

### TB care and treatment in South Fly District

The Western Provincial Health Authority (WPHA) led the TB response and programme and was supported by implementation partners (Burnet Institute for TB Technical and Implementation Support and Health Systems Strengthening; World Vision for Community Engagement and Care). TB diagnosis is centralised at the DPH TB diagnostic centre, where patients can present themselves, are referred from community sites, or are referred from other hospital units. People with presumptive TB are registered and asked to provide two sputum samples for testing with Xpert^®^ MTB/RIF (Cepheid, Sunnyvale, CA, USA), the initial test since 2016, in addition to chest radiography (CXR). Paediatric patients who are unable to produce sputum and those with signs or symptoms of extrapulmonary TB may undergo further procedures – gastric aspirate, fine-needle aspiration of lymph nodes – for testing using WHO-recommended molecular rapid diagnostic tests. In 2019, Xpert^®^ Ultra (Cepheid) replaced Xpert MTB/RIF, and in April 2022, Xpert XDR was introduced at the Central Public Health Laboratory (CPHL) in Port Moresby. All samples with MTB detected and rifampicin-resistant are sent to the CPHL for culture and drug susceptibility testing (C-DST). However, C-DST has been unavailable at CPHL since 2020 as the laboratory was not functional. A small number of C-DST samples were sent to the supranational reference laboratory in Australia from early 2022.^13^

DPH was the first site in PNG to systematically introduce the WHO Group A and B MDR-TB drugs, enabling a long all-oral regimen with the corresponding phase-out of injectable agents from 2016.^17^ People with TB are managed according to the national TB protocols and WHO guidelines. Previously, regimens for MDR/RR-TB have included four potential standardised regimens:^18^ three long (18–24 months) regimens that could consist of an injectable agent or newer TB drugs (bedaquiline, delamanid) and a shorter (9–12 months) regimen that included an injectable agent. Patients with pre- or extensively drug-resistant TB (XDR-TB) were treated with longer regimens.

TB treatment initiation is ambulatory unless patients need admission for medical or social reasons. Treatment delivery is via regular support from community health workers and supporters at community treatment sites in Daru. A standard model of care for MDR/RR-TB treatment and care developed in Daru provides patient support with directly observed therapy where possible, as per the national protocol. Since 2016, patient-centred care has been provided for DR-TB in Daru, with patient education and counselling, daily meals, adverse event management and referral.^19^ For patients residing outside Daru, treatment is initiated at Daru BMU, but self-administered MDR/RR-TB treatment may be provided for periods for select patients who can be monitored by a family or community treatment supporter and return monthly for clinical review.

### COVID-19 in PNG

**FIGURE 1.**
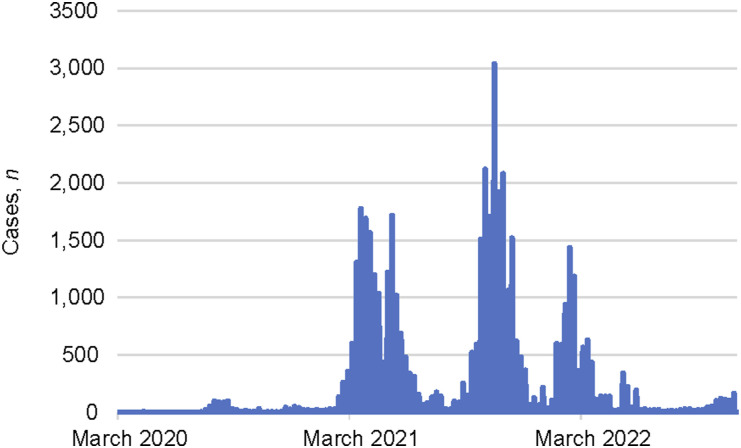
COVID-19 notifications in Papua New Guinea, 2020–2022. Source: Based on World Health Organization. Population based on various sources. Geneva, Switzerland: WHO, 2024; adapted by Our World in Data.

PNG experienced a small wave of COVID-19 in 2020 and three major waves of reported COVID-19 cases and deaths from 2021 to 2022, as displayed in Figure 1.^12^ As of September 2023, when routine reporting stopped, there were over 47,000 confirmed COVID-19 cases and 670 deaths reported.^20^ However, this underestimates the actual burden in PNG due to minimal testing, contact tracing and underreporting.^20^ Testing rates were persistently low due to barriers such as misinformation, lack of community trust, limited access, and logistic and supply issues.^21^

Health system strain was reported, with hospitals overwhelmed across the second and third waves. A state of emergency was declared on 23 March 2020, involving international border closures, quarantine, a National Pandemic Act, and international resource mobilisation. In the first 100 days, three nationwide lockdowns of up to 2 weeks were instituted where health services only opened to critical and emergency services.^12^ After this time, only localised lockdowns were implemented, which were less significant in scale or duration. In August 2020, the ‘Niupela Pasin’ prevention and mitigation strategy was introduced, emphasising face masks, social distancing and hand hygiene. Isolation and quarantine were generally not enforced, and community uptake of public health measures was generally very low, with variations across the provinces. COVID-19 vaccination commenced in March 2021, but coverage rates were very low; 7.1% of the population received a single dose by the end of 2022.^12^ In February 2022, government policy shifted to a reduction in public health measures and testing and international borders were re-opened.

### Study definitions

Programmatic interventions, TB notifications, and outcomes were compared between ‘pre-COVID’ (1 January 2017–31 December 2019) and ‘COVID’ (1 January 2020–31 December 2022). Annual cohorts were used to note the COVID-19 impacts that commenced in March 2020.

The study definitions used for TB registration and outcomes were per the 2022 WHO definitions^18^ and most recent PNG TB guidelines (2017), noting the evolution of DR-TB reporting definitions over time.^22^

### Data collection and analysis

At Daru BMU, TB cases are recorded in paper-based programmatic registers and an electronic medical record system (EMR) (Bahmni v.0.87; Thought Works, Chicago, IL, USA). Data were exported from the TB treatment registration dataset and cleaned and analysed using STATA v17 (Stata Corp, College Station, TX, USA). By annual cohort, numbers and proportions were calculated for notified cases and outcomes. Statistical significance between two proportions was tested using standard error.

Annual TB case notification rates were calculated based on population data from 2021 estimates, with residential addresses provided at registration coded to census units.^15^ Programmatic activities and interventions were grouped using a conceptual framework based on the objectives in the WPHA TB annual implementation plan – TB model of care (case-finding, treatment and care, prevention), health systems (governance, human resources, financing), community engagement and data utilisation and research. COVID-19 interventions were described in narrative format.

### Ethics

This study received ethical approval from the PNG Medical Research Advisory Committee, Port Moresby, PNG.

## RESULTS

**FIGURE 2.**
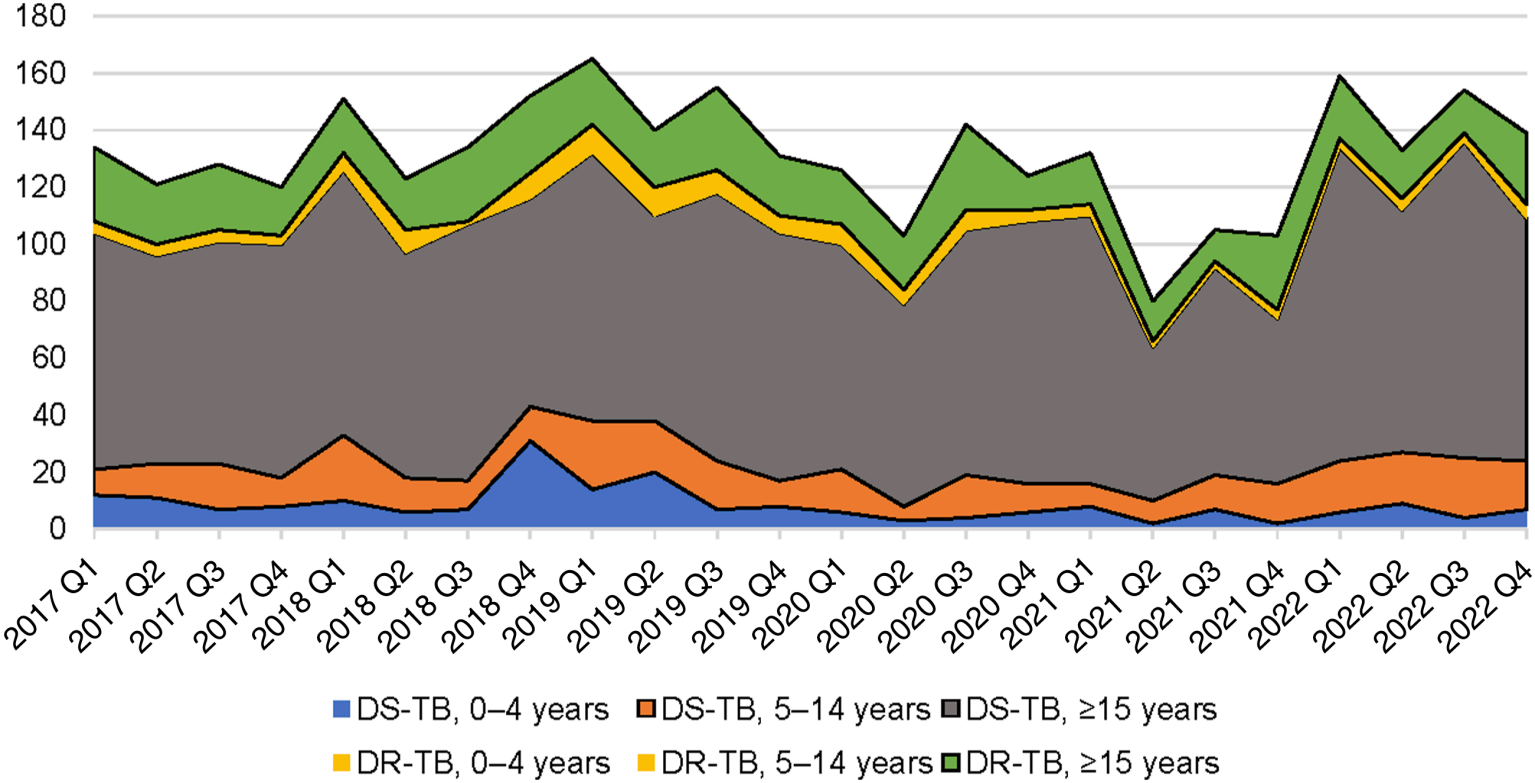
TB registrations in South Fly District, PNG, 2017–2022, by age and drug resistance. Q = quarter; DR-TB = drug-resistant TB; DS-TB = drug-susceptible TB; PNG = Papua New Guinea.

There were 3,156 people registered for TB in the study period: 1,656 (52.5%) pre-COVID and 1,500 (47.5%) in the COVID period. Figure 2 shows TB registration data by year disaggregated by age and drug resistance. Table 1 displays the clinical and demographic characteristics of people registered with TB from 2017 to 2022 in SFD. There was a 29.2% reduction in case notifications from 2019 to 2021, with recovery in 2022, except for the 0–4-year age group. This disruption was greater in DR-TB (37.0%) than DS-TB (27.0%). Case notification per 100,000 people for SFD was 623 over the entire study period, decreasing by 9.5% from pre-COVID to COVID-19. The case notification rate was 1,792 per 100,000 for Daru residents over the study period.

**TABLE 1.** Clinical and demographic characteristics of people with TB in South Fly District, 2017–2022.

Characteristic	2017–2019 Pre-COVID *n* (%)	2020–2022 COVID *n* (%)	Total *n* (%)
Total people with TB	1,656	1,500	3,156
Median age, years	25	25	25
Age group, years			
0–4	166 (10.0)	79 (5.3)	245 (7.8)
5–14	220 (13.3)	195 (13.0)	415 (13.1)
15–34	735 (44.4)	741 (49.4)	1,476 (46.8)
35–54	390 (23.6)	396 (26.4)	786 (24.9)
≥55	143 (8.6)	89 (5.9)	232 (7.4)
Missing	2 (0.1)	0 (0.0)	2 (0.1)
CNR, /100,000 population			
SFD (all TB)	654	592	623
SFD (DR-TB)	135	109	122
Daru (all TB)	1,806	1,779	1,792
Female sex	795 (48.0)	694 (46.3)	1,489 (47.2)
Primary residence			
Daru	1,051 (63.5)	1,035 (69.0)	2,086 (66.1)
Outside Daru	603 (36.4)	465 (31.0)	1,068 (33.8)
Unknown	2 (0.1)	0 (0.0)	2 (0.1)
South Fly District	1,597 (96.4)	1,448 (96.5)	3,045 (96.5)
Other or Unknown	59 (3.6)	52 (3.5)	111 (3.5)
HIV status			
Negative	1,484 (89.6)	1,390 (92.7)	2,874 (91.1)
Positive	45 (2.7)	31 (2.1)	76 (2.4)
Unknown	127 (7.7)	79 (5.3)	206 (6.5)
Registration			
New	1,469 (88.7)	1,339 (89.3)	2,808 (89.0)
Previously treated	176 (10.6)	152 (10.1)	328 (10.4)
Unknown	11 (0.7)	9 (0.6)	20 (0.6)
Disease site			
Pulmonary	1,075 (64.9)	904 (60.3)	1,979 (62.7)
Extrapulmonary TB	581 (35.1)	595 (39.7)	1,176 (37.3)
Unknown	0 (0.0)	1 (0.1)	1 (0.0)
Diagnosis			
Bacteriologically confirmed	1,026 (62.0)	1,081 (72.1)	2,107 (66.8)
Clinically diagnosed	630 (38.0)	411 (27.4)	1,041 (33.0)
Unknown	0 (0.0)	8 (0.5)	8 (0.3)
Resistance			
Drug-susceptible TB	1,313 (79.3)	1,223 (81.5)	2,536 (80.4)
Drug-resistant TB	343 (20.7)	277 (18.5)	620 (19.6)
Mono/polyresistance	1 (0.1)	1 (0.1)	2 (0.1)
MDR-TB	209 (12.6)	81 (5.4)	290 (9.2)
Rifampicin resistance	96 (5.8)	174 (11.6)	270 (8.6)
Pre-XDR-TB	15 (0.9)	13 (0.9)	28 (0.9)
XDR-TB	14 (0.8)	2 (0.1)	16 (0.5)
Not recorded	8 (0.5)	6 (0.4)	14 (0.4)

CNR = case notification rate; SFD = South Fly District; MDR-TB = multidrug-resistant TB (excluding pre-XDR and XDR-TB); XDR-TB = extensively drug-resistant TB.

Among registered people with TB, half (53%) were male, with a median age of 25 years (interquartile range [IQR] 17–40), 20.9% were children (<15 years), 66.1% resided in Daru, and 96.5% were from SFD. Of all people with TB, 66.8% were bacteriologically confirmed (15.5% were under 15 years old), 10.4% were re-treatment, 58.9% were registered as pulmonary TB and 19.6% as DR-TB. HIV infection prevalence was 2.2%; 10% of the cohort had unknown status.

**FIGURE 3.**
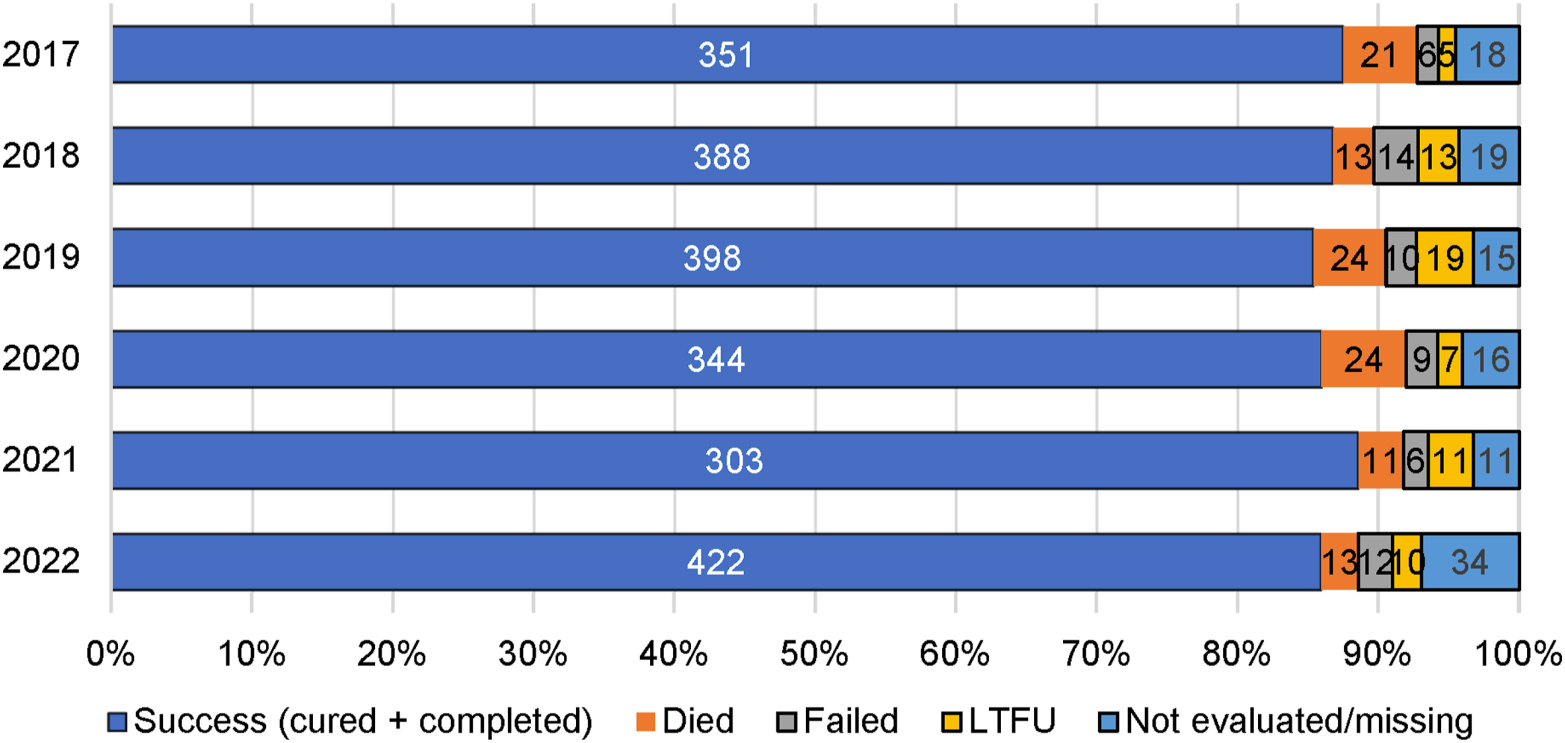
Treatment outcomes for drug-susceptible TB from 2017 to 2022 in South Fly District, PNG. LTFU = loss to follow-up; PNG = Papua New Guinea.

**FIGURE 4.**
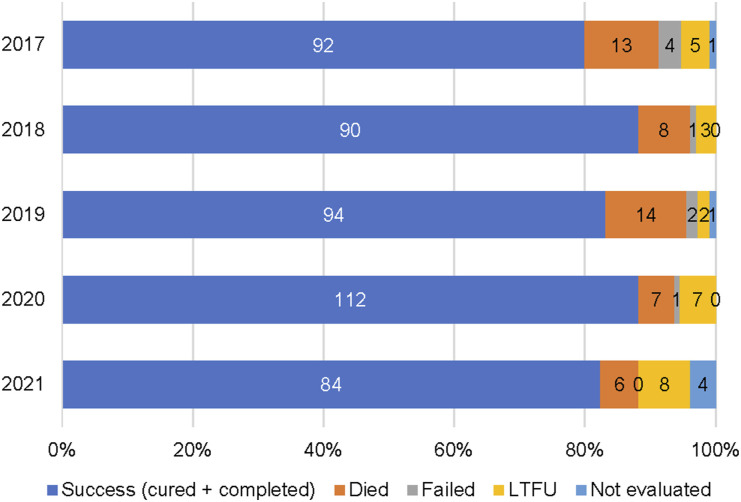
Treatment outcomes for drug-resistant TB from 2017 to 2021 in South Fly District, PNG. LTFU = loss to follow-up; PNG = Papua New Guinea.

Compared to the pre-COVID period, in the COVID period, there were statistically significant but small increases in residence in Daru and bacteriological confirmation and decreases in TB notifications in young children (0–4 years), pulmonary TB and DR-TB. An increase in RR-TB offset the decrease in MDR-TB in COVID. Pre-XDR and XDR-TB did not change significantly. The overall treatment success rate was 86.5% for DS-TB and 83.6% for DR-TB. Treatment success rates, TB deaths and loss of follow-up did not change significantly during COVID (Figures 3 and 4).

Table 2 describes the programmatic interventions for TB in the pre-COVID and COVID periods. There were impacts on TB services through 2020–2021, with a return to pre-COVID activities in 2022. During the COVID-19 period in SFD, the duration of lockdowns was limited to the initial national period in 2020. Population movement may have been reduced with the reduction in domestic travel and border closures to the Torres Strait (from March 2020 to late 2022). There was a notable and significant impact on the health workforce, including increased absenteeism (isolation and quarantine during waves), diversion of staff to the COVID-19 response, and reduced services. During the COVID period, there was an observed increase in community mask use and significant community engagement and health promotion on prevention measures. Compared with other provinces, there was a concerted vaccination rollout in SFD and the PNG-Australia treaty villages with moderate uptake.

**TABLE 2.** Programmatic interventions undertaken as part of the COVID-19 response in SFD, Western Province, PNG.

Strategic interventions	Pre-COVID 2017–2019	COVID 2020–2022
1. Establish a model of patient-centred TB care for diagnosis, treatment and prevention of DS-TB and MDR-TB at Daru		
Diagnosis, treatment, care and prevention	Scale up of routine household contact investigation from MDR-TB index cases to include DS-TB index cases and TPT for young child contacts (<5 years) Systematic screening initiative commenced with chest X-ray with CAD4TB to detect active TB in Daru residents ≥10 years old (2018) BCG assessment following stockouts and low coverage in 2018: BCG coverage in SFD 64% (2017), 21% (2018), 48.4% (2019)	Model of care was scaled back to essential services to focus on treatment and care, and no active case-finding or TPT in 2020–2021 Flexible treatment delivery with self-administered treatment Facility-based TB detection (passive case-finding) was constrained due to a lack of clinical staff COVID-19 training and bidirectional COVID-19 and TB screening and diagnosis; COVID-19 education to TB patients and general community support to COVID-19 vaccination engagement and delivery programme BCG coverage improvement from 40.9% (2020) to 27.1% (2021) and 66.1% (2022)
2. Strengthen health systems building blocks to enable a functional model of TB care		
Governance	Regular TB programme and partner coordination meetings	Disruption to routine TB coordination meetings and TB annual implementation plans not reviewed in 2020-21
Human resources	Continued partnership model of international technical support from Burnet: training, development of SOPs, on-the-job mentoring, task-shifting	Disruption to key TB service delivery clinical and public health roles Demobilisation from PNG of international technical advisers (2020–2021) and remote support commenced. PNG TB clinical fellowship program commenced, rotating clinicians from other provinces to Daru (2021)
Information systems, supply chain, and laboratory systems	Introduction of electronic medical records system (EMRS) for DR-TB care and programmatic management	Significant stockouts: PPE, TB drugs (resolved with supplementary orders), Xpert Ultra (2020–2021) National TB laboratory for C-DST was not functional (2020–2021)
3. Improve service utilisation and TB prevention through community engagement		
Community engagement	TB survivors engaged through the PEC programme TB patient representative group formed Community consultations for community-wide screening and prevention conducted, and CAG formed	CAG and patient representative meetings disrupted until 2022 Model of community engagement developed for TB applied to COVID-19 Household engagement for community-wide screening (2022)
4. Programme data are utilised for effective action		
Data utilisation and operational research	Real-time TB care cascades (EMRS) for programme improvement Structured Operational Research Training Initiative (SORT-IT) conducted in Daru and course completed	TB programme weekly process indicator reporting commenced to track service utilisation for detection and retention in care and prevention All operational research on hold Planned second SORT-IT delayed until 2022
5. Decentralised TB care is established in SFD		
Decentralise care and treatment	Health facility and needs assessments for decentralisation, with plans developed Training conducted at two decentralised sites outside Daru (Mabadawan and Balimo)	Limited progress on plans for decentralisation of TB services outside Daru and province-wide (2020–2021) New health centre at Mabudawan opened in 2020

SFD = South Fly District; PNG = Papua New Guinea; DS-TB = drug-susceptible TB; MDR/RR-TB = multidrug and rifampicin-resistant TB; TPT = TB preventive therapy; CAD4TB = Computer-Aided Detection for Tuberculosis; BCG = bacille Calmette-Guèrin; SOP = standard operating procedures; PPE = personal protective equipment; CAG = community advisory group; PEC = patient education and counselling.

## DISCUSSION

There are very high TB case notifications in SFD with alarming levels of incident TB in Daru residents (1.8%). TB case detection declined by 29% in 2021 compared to pre-COVID, with recovery in 2022. Despite COVID-related disruptions to TB services, successful TB treatment outcomes were maintained. The interventions and resources directed towards TB and health systems strengthening in Daru and SFD since 2015 have likely contributed to this and may have included - the formation of effective and sustained partnerships and enabling governance mechanisms, the availability of technical assistance during the pandemic, a focus on community engagement, training and development of capacity in the community and health workforce, the implementation of standardised procedures for facility and community-based TB and DR-TB care, and a focus on data management through electronic reporting and data utilisation to inform decision-making.

This study is the first report of TB detection and treatment outcomes during the COVID-19 pandemic in PNG. A strength of the study is the data quality due to the use of the EMR, which was not significantly disrupted during COVID, allowing enhanced TB reporting compared with many similar remote settings. A limitation of the study was that it did not capture COVID-19 surveillance and programmatic activities, nor did it include TB data in SFD, thereby restricting the scope of interpretation. This is a descriptive study with limited information to explain the findings, and therefore, the interpretation is speculative.

The increase in bacteriological confirmation during COVID demonstrates that laboratory diagnostic services remained functional, with a likely reduction in the clinical diagnosis of TB due to human resources shortages. The data from SFD reflect global trends of disruptions to TB services from COVID-19, with an 18% reduction in TB notifications in 2020, partial recovery in 2021 and 2022 levels rebounding to above those in 2019.^1^ The Western Pacific had the largest reduction and smallest recovery of all regions. Globally, it was estimated that TB incidence increased by 3.9% from 2020 to 2022, and TB-related deaths increased from 2020 to 2021.^1^ This reflects the impact of disruptions to access and provision of TB services during the COVID-19 pandemic that led to more undetected TB and increased transmission.

In SFD, COVID-19-related changes in the health workforce and health system had a major impact on TB services. Active case finding among household contacts was halted, and community access and utilisation of TB diagnostic services were reduced despite the limited duration of lockdowns in SFD. The reduction in TB detection in young children is consistent with observations elsewhere.^23^ This may reflect an increased detection gap because of interruption to facility-based services for clinical diagnosis and household screening of child contacts. As TB in young children is a sentinel marker for recent community transmission, it may also indicate less exposure to TB during the COVID period, at least outside of the household setting.

Globally, TB treatment success for MDR/RR-TB dropped to 63% in 2020 and increased to 88% in 2021.^1^ That treatment outcomes did not fall in SFD, a remote and resource-limited setting, was likely due to the health systems strengthening activities that had occurred since 2015 and the targeting of the reduced resources on patient care and support – including education, counselling, nutritional support and flexible delivery of care. The peer counselling model that continued during COVID-19 may have been influential – treatment adherence challenges were identified in 43% of 16,234 counselling sessions over the study period.^19,24^ People on treatment were supplied TB medications to take home (self-administered treatment), including for DR-TB. The program-initiated process indicators were monitored to track the weekly proportion of patients retained in care, and community health worker teams were engaged in follow-up and support. An examination of lessons from TB and COVID-19 response in Brazil, India and South Africa noted key interventions that can be deployed to address both TB and COVID-19 – behavioural prevention methods, including mask-wearing, screening and contact tracing, digital and mHealth technologies, multi-sectoral engagement and community-led participation.^25^

Operational research from similar low-resource, high TB burden settings has described the impact of the pandemic. In Malawi and Sierra Leone, there was a reduction in TB case detection and maintenance of successful treatment outcomes during COVID-19.^26,27^ In other settings such as Brazil, Kazakhstan, Mexico and Zimbabwe, treatment success decreased during the COVID-19 period to early 2021.^28–31^ A health systems strengthening and TB quality improvement project in Mimika District, Papua Province, Indonesia demonstrated greater resilience to COVID-19 impacts than reported nationally.^32^ The experience in this remote, high-incidence setting was not dissimilar to our experience across the border in SFD. There was a drop in annual case notifications by 19%, but treatment outcomes were maintained, and a rapid ‘recovery’ of detection was noted. Compared with control clinics, a health system strengthening intervention in South African primary health care clinics demonstrated increased TB testing and detection.^33^

There remains an alarming TB notification rate in Daru from 2014 to 2022, with 20% of the burden DR-TB and likely substantial ongoing transmission evidenced by the persistently high rates of case detection since 2014 and a high proportion of TB in children, adolescents and young adults. Most TB notifications are in Daru Island residents, which did not change over time. However, there may be limitations in recording this information (e.g., patients may have cited residence in Daru because they have temporarily relocated to seek health care). Resources, diagnostic services, and community awareness activities have continued focusing on Daru over time, with limited decentralisation; therefore, the burden in wider SFD remains unknown.

Strengthening the TB and health systems in SFD has likely contributed to successfully maintaining TB detection and treatment outcomes during COVID-19. However, high levels of community transmission remain, and additional interventions are urgently needed to address this. As the health system impacts of the pandemic have declined, the planned community-wide screening and prevention of TB recommenced in Daru in 2023.^9^ COVID-19 had a devastating impact in PNG, like many low- and middle-income countries, and has limited progress on TB. The experience in SFD with a long-term (10-year) program of TB technical assistance, coupled with health systems strengthening, has provided resilience to this shock to a degree. There is a need to strengthen health systems as part of TB programs to enhance preparedness for future health threats.^34,35^

## Supplementary Material


