# Supplementary material for: TB programme outcomes in South Fly District, Papua New Guinea, were maintained through COVID-19
Source: Public Health Action. 2024 Dec 1;14(4):139–45. doi: 10.5588/pha.24.0020 (PMC11604148; doi:10.5588/pha.24.0020)
Supplement: Supplementary file 1 [file pha24-0020_supplementarydata1.docx]

# TB programme outcomes in South Fly District, Papua New Guinea, were maintained through COVID-19

**SUPPLEMENTARY DATA**

**Table 2 - Detailed Version**

**Programmatic interventions undertaken for the COVID-19 response in South Fly District, Western Province, Papua New Guinea**

| **Strategic Interventions** | **Pre-COVID**  **2017-2019** | **COVID**  **2020-2022** |
| --- | --- | --- |
| **1. Establish a model of patient-centred TB care for diagnosis, treatment and prevention of DS and MDR-TB at Daru** | | |
| Diagnosis, Treatment, Care and Prevention | 2017   - Introduction of shorter treatment regimens for MDR/RR-TB (9 months, containing injectable agent). Expansion of use of the oral, injectable free long MDR/RR-TB regimen with increasing use of bedaquiline (45% of treatment initiations in 2017 compared with 19% in 2016) - Strengthened community treatment sites and support for patient monitoring (side-effects, referral, sputum collection) - Psychosocial support with standardised patient education and counselling for DR-TB and DS-TB patients, including referral to family planning, family sexual violence and disability services - Child TB initiatives including updated guidelines, training, child-friendly education, counselling - Scale up of routine household contact investigation from MDR-TB index cases to include to DS-TB index cases (1384 contacts screened) - Routine TB preventive therapy (TPT) for DS-TB for children under 5 (38 initiated TPT) - Infection control interventions at DPH including ventilation assessments, triage, respirator fit testing   2018   - Systematic screening initiative commenced with chest x-ray with CAD4TB (a single fixed central location on a truck) to detect active TB in Daru residents 10 years and above (led by WHO) - Contact investigation expanded to DS-TB index cases   2019   - Systematic screening initiative completed early 2019 with modest yield (69 additional cases, <1% of those screened), compared with 2.5% in contact screening - TB program plans developed for a comprehensive search-treat-prevent model in Daru community (for 2020), ie expanding TPT to older children and adults, given limited yield of active case finding alone. - Expanded TPT to young child contacts (under 5 years) of MDR-TB index cases - Introduction of new TPT regimens for young child contacts: 3RH and 6Lfx - Dedicated peer counsellor for TPT program - Xpert Ultra replaced Xpert MTB/RIF - BCG assessment following stockouts and low coverage in 2018: BCG coverage in SFD 64% (2017), 21% (2018), 48.4% (2019) | 2020   - With the onset of the pandemic, the model of care was scaled back to essential services to focus on treatment and care - Adaptation of model of care to allow flexible treatment delivery with self-administered treatment for longer periods. New SOPs developed for community-based care (patient triage, sputum transport, infection prevention, care and support) - PEC services reduced from March to June, but then resumed as normal for active TB and TPT - Facility based TB detection (passive case funding) was constrained due to lack of clinical staff. Reduced attendances to the TB diagnostic centre at DPH was recorded. - Active case finding, including household contact investigation and initiation of new patients on TB preventive treatment (TPT) was stopped in March. - Plans for population screening were deferred. Household contact investigation resumed in November. - No change to treatment regimens - COVID-19 training conducted for clinicians, SOPs developed including for IPC, PPE and bidirectional COVID-19 and TB screening and diagnosis - COVID-19 planning and response framework developed with WPHA - COVID-19 education to TB patients and general community   2021   - TPT provision for young child contacts resumed. Weekly clinical reviews decentralised from DPH to community sites. - Support to COVID-19 vaccination engagement and delivery program   2022   - Return to full TB program activities including routine household contact screening (February), TB diagnostic services and outreach clinics. - Xpert XDR introduced at the central public health laboratory (CPHL) - BCG coverage improvement from 40.9% (2020), 27.1% (2021) to 66.1% (2022) - COVID vaccination education flipchart adopted nationally |
| **2. Strengthen health systems building blocks to enable a functional model of TB care** | | |
| Governance and coordination | 2017   - Regular TB program coordination meetings with WPHO and partners (Burnet, World Vision) - Coordination with National Emergency Response Taskforce (ERT)   2018   - Partner monitoring framework established   2019   - Provincial Health Office (public and community health) and DGH (curative, reporting to NDOH) transitioned to WPHA in September 2019 | 2020   - New WPHA structures announced and interim executive commenced in Q3 and WPHA building its capacity and operational functions - Disruption to routine TB coordination meetings, although weekly partnership meetings held for COVID-19 where TB issues were discussed - WPHA TB annual implementation plans not reviewed   2021   - Full WPHA executive positions appointed but governance and program functions still impacted . - WPHA annual implementation plan reviewed. Ongoing disruptions to some routine coordination meetings.   2022   - Return to routine governance meetings and joint planning. |
| Financing | - Australian Government and World Bank/NDOH Emergency TB Project commenced to support systematic screening initiative (2017) - Additional funding obtained for operational research projects via Australian government research funding, aiming to strengthen data utilisation, expand contact screening and management and molecular epidemiology as operational research (2018-19) | No disruption to TB financing but no increases |
| Human resources, capacity building & technical assistance | 2017   - Continued partnership model of technical support including international specialists and medical officers through Burnet, supporting care and treatment and community-based screening and prevention - Telemedicine case discussions with international experts - Training, task-shifting and development and updating of SOPs - Contact tracing and TPT teams established without additional resources - Provincial TB coordinator (leadership) position remained unfilled with an acting role - Gaps in clinical TB roles, filled by short term international advisors   2018   - Workforce assessment by WHO - Competency framework for TB workforce capacity building developed, following a training needs assessment   2019   - TB physician appointed - TB human resources review and mapping | 2020   - Key roles in the WPHA unfilled due to roles in COVID response - Disruption to key TB service delivery clinical and public health roles - Demobilisation from PNG of international technical advisers for clinical and public health TB activities (including service delivery support) from 2020 to early 2021. Locally engaged staff remained. Remote support enhanced – included training, monitoring and case discussions / troubleshooting. - Significant ongoing gaps in WPHA and DPH clinical TB roles - PNG TB clinical fellowship program commenced, rotating clinicians from other provinces to Daru.   2021   - WPHA provincial disease control and TB officer appointed   2022   - Integration of partner and WPHA roles into endorsed TB program human resources structure - Peer counselling program ongoing with child protection, child counselling training, TB/HIV training and exchanges with - Commencement of SWEEP-TB (comprehensive community screening and prevention project) in November. |
| Information systems, supply chain, & laboratory systems | 2017   - Electronic medical records system (EMRS) for DR-TB care and programmatic management in SFD implemented – open source (Bahmni) - Review of stock management and procurement systems for community sites and establishment of local storage facilities   2018   - Expansion of EMRS to include DS-TB treatment, PEC and contact screening and management   2019   - TB data analytics site for WPHA with dashboards developed from EMRS for real-time reporting | 2020   - Significant stockouts – PPE, TB drugs (resolved with supplementary orders), Xpert Ultra - National TB laboratory for C-DST was not functional and testing not performed - TB program office requiring repairs and unable to accommodate staff   2021   - Ongoing stockouts of consumables, Xpert Ultra and some MDR-TB drugs (supplementary orders). Temporary disruption to the EMRS - Additional TB program office space secured   2022   - Implementation of an electronic logistic management information system for non-TB supplies at DPH. Resolution of most stockouts (except cycloserine). Resumption of national TB lab services, including C-DST at CPHL. Xpert XDR available at DPH. |
| **3. Improve service utilisation and TB prevention through community engagement** | | |
| Community engagement | 2017   - TB survivors engaged through PEC program - Community workshops on community mobilisation and advocacy - TB patient and community needs assessment conducted to inform support model   2018   - TB patient representative group formed and patient satisfaction surveys conducted   2019   - Photovoice activity to document lived experience with TB in Daru, strengthen peer support and build community awareness - Community consultations for community-wide screening and prevention conducted, with agreement to form CAG | 2020   - First meeting of the CAG for the community-wide screening project (prior to COVID) with plans for community engagement developed - Model of community engagement developed for TB applied to COVID-19 - Integration of community engagement and education for TB an COVID - TB education and counselling Facebook page launched and included COVID-19 information   2021   - CAG meetings resumed with terms of reference and self determined name: *TB Nanito Kopia Kodu Group* (the Voice to Kill TB Forever)   2022   - Household engagement, mapping and design and planning for community-wide screening. Patient representative meetings restarted. CAG provided input into screening program design. Community education materials developed for SWEEP-TB (including TPT for adolescents and adults) |
| **4. Program data is utilised for effective action** | | |
| Data utilisation and operational research | 2017   - EMRS used to establish TB care cascades for program improvement - TB research prioritisation and agenda conducted - Structured Operational Research Training Initiative (SORT-IT) conducted in Daru - Molecular epidemiology of DR-TB in Daru research conducted to inform treatment regimens and public health responses - Geographic reconnaissance survey conducted in Daru to quantify households and residents   2018   - Socio-behavioural research on case and contact perspectives of TB care conducted - 4 SORT-IT studies from SFD completed and presented at PNG medical symposium - Quality improvement initiative conducted on sputum collection for patient monitoring (increased from 20% to 82%) - Modelling and cost-effectiveness analysis of TB interventions in Daru to inform strategy and planning   2019   - TPT operational research commenced including TPT for young child contacts of MDR-TB index cases - Publication of 12 operational research articles (4 from SFD) from SORT-IT in journal supplement (Public Health Action) | 2020   - TB data analytics dashboards developed for program decision makers - TB program weekly process indicator reporting commenced to track service utilisation for detection and retention in care and prevention - All operational research on hold. Planned SORT-IT course cancelled   2021   - Operational research protocol for SWEEP-TB approved by ethics review boards in PNG and Australia - Contributed to a national study on the impact of COVID-19 on essential services, including TB   2022   - National SORT-IT TB course conducted with 3 projects from WPHA - Outbreak investigation conducted for an increase in detection of fluoroquinolone resistant TB |
| **5. Decentralised TB care is established in SFD** | | |
| Decentralised  care and treatment | 2017   - Health facility and needs assessments for decentralisation, including survey of DR-TB patient   2018   - Decentralisation plans developed to identify 2 priority TB BMUs outside Daru – Mabadawan and Balimo, where trainings were conducted.   2019   - Construction of Mabadawan health centre on Torres Strait border. - Partner (PNG sustainable development program, SDP) engaged in Balimo to strengthen health centre - Aviation clinical outreach visits commence across province via SDP for primary care (not TB specific) | - Limited progress on plans for decentralisation of TB services outside Daru and province wide.   2020   - Planned visits cancelled. New Mabadawan health centre opened   2021   - Education and counselling team conducted training and support visits to decentralised sites, introducing standardised PEC in Balimo. - GeneXpert set up in Balimo   2022   - Resumption of outreach clinics and training for TB care and support to Balimo. Facility assessment conducted at Mabudawan. |
| WPHA - Western Provincial Health Authority; SFD – South Fly District; DPH - Daru Provincial Hospital; NDoH - National Department of Health; MDR/RR-TB – multidrug and rifampicin resistant TB; XDR – extensively drug-resistant TB; PEC - patient education and counselling; CAG - Community advisory group; TPT – TB preventive treatment; H – isoniazid; R – rifampicin; Lfx – Levofloxacin; SORT-IT - Structured Operational Research Training Initiative; SOP – Standard operating procedures; PPE – personal protective equipment; IPC – infection prevention and control; EMRS – electronic medical record system; WHO – World Health Organisation; SWEEP-TB – Systematic community-wide engagement and elimination project for TB in Daru. | | |
